# Prognostic Factors and Treatment Outcomes in Gallbladder Cancer Patients Undergoing Curative Surgery: A Multicenter Retrospective Cohort Study

**DOI:** 10.3390/curroncol32060328

**Published:** 2025-06-03

**Authors:** Bowen Xu, Yanjiang Yin, Jianping Chang, Zhiyu Li, Xinyu Bi, Jianqiang Cai, Xiao Chen

**Affiliations:** 1 Department of Hepatobiliary Surgery, National Cancer Center/National Clinical Research Center for Cancer/Cancer Hospital, Chinese Academy of Medical Sciences and Peking Union Medical College, Beijing 100021, China; xubowen1112@126.com (B.X.); bruceyin@student.pumc.edu.cn (Y.Y.); chang_jianp123@126.com (J.C.); lizhiyu@cicams.ac.cn (Z.L.); bixinyu@cicams.ac.cn (X.B.); 2 Department of Hepatobiliary Surgery, General Surgery, Qilu Hospital, Cheeloo College of Medicine, Shandong University, Jinan 250012, China; 3 Beijing Key Laboratory of Cell and Gene Therapy for Digestive System Cancers, National Cancer Center/National Clinical Research Center for Cancer/Cancer Hospital, Chinese Academy of Medical Sciences and Peking Union Medical College, Beijing 100021, China; 4 Department of Gastrointestinal Surgery, Beijing Tsinghua Changgung Hospital, School of Clinical Medicine, Tsinghua University, Beijing 102218, China

**Keywords:** gallbladder carcinoma, curative surgery, overall survival, prognostic biomarker, surgical outcomes

## Abstract

Background: Gallbladder cancer (GBC) is a highly aggressive malignancy often diagnosed at an advanced stage due to its asymptomatic onset. Despite surgery being the only potentially curative option, recurrence and poor prognosis remain common, especially in advanced-stage diseases. There is limited consensus regarding the extent of lymphadenectomy, hepatic resection, and the role of adjuvant therapies. Identifying prognostic factors and optimizing treatment strategies are critical for improving outcomes. This multicenter retrospective study was conducted to evaluate the clinical and pathological predictors of survival and recurrence in GBC patients that underwent radical surgery and to assess the potential benefit of adjuvant therapies in advanced stages. Methods: This was a retrospective cohort study of GBC patients who underwent curative-intent resection for GBC between 2010 and 2022 at two tertiary medical centers in China. The baseline characteristics, surgical data, pathology, adjuvant therapy, and follow-up outcomes were analyzed. The survival outcomes were assessed using Kaplan–Meier methods and Cox regression models. Subgroup analyses were conducted to explore the impact of postoperative adjuvant chemotherapy, period of surgical treatment, and extent of resection. Multiple imputation was used to address missing data. Results: The 5-year overall survival (OS) rate was 57.4%. Independent predictors of a poorer OS included CA19-9 > 30 U/mL (HR = 1.861, *p* = 0.003), poor/moderate-to-poor differentiation (HR = 2.134, *p* = 0.004), T3–T4 stage (HR = 2.685, *p* = 0.001), N1–N2 stage (HR = 2.217, *p* = 0.002), M1 stage (HR = 2.308, *p* = 0.001), and a high CAN score (HR = 1.875, *p* = 0.009). Adjuvant chemotherapy improved the OS in the stage III–IV patients (24.8 vs. 17.3 months, *p* = 0.036), though the DFS improvement was not significant (*p* = 0.133). No survival difference was observed between the segment IVb + V resection and wedge resection in the T2b patients. The patients treated after 2017 had a better OS (*p* = 0.024), possibly due to improved surgical techniques and perioperative care. Conclusions: Radical surgery remains critical for GBC. Accurate staging and tailored perioperative strategies, including chemotherapy, may improve outcomes, though further prospective studies are needed to validate these findings.

## 1. Introduction

Gallbladder cancer (GBC) is the most common malignancy of the biliary tract and a major cause of cancer-related mortality, particularly in regions with a high prevalence of gallstone disease, such as South Asia and Latin America [1,2]. The disease often presents at an advanced stage due to its asymptomatic nature in the early phases, contributing to a poor prognosis and limited treatment options [3]. In China, the incidence of GBC has shown a gradual increase, reflecting changes in diet, lifestyle, and improved detection [4].

Curative-intent surgical resection remains the cornerstone of treatment for early-stage GBC and offers the only potential for long-term survival. However, the recurrence rates remain high, especially in advanced-stage disease, underscoring the need for improved treatment strategies [5,6]. In recent years, there has been progress in refining surgical techniques (e.g., extent of liver resection and lymphadenectomy); perioperative management; and the integration of systemic therapies, including adjuvant chemotherapy and targeted agents [7,8].

In recent years, there have been advances in understanding the clinical and pathological factors that influence GBC prognosis. Multiple studies have identified the tumor stage (T, N, and M classification), histologic grade, lymphovascular and perineural invasions, serum CA19-9 level, inflammatory markers (e.g., neutrophil-to-lymphocyte ratio), and extent of resection as significant predictors of survival and recurrence [5,9,10]. Additionally, the number of lymph nodes resected and the margin status (R0 vs. R1/R2) are strongly associated with long-term outcomes [11].

Notably, the BILCAP trial established capecitabine as the standard adjuvant chemotherapy in resected biliary tract cancers, including GBC, leading to its incorporation into the National Comprehensive Cancer Network (NCCN) and European Society For Medical Oncology (ESMO) guidelines [12,13]. More recently, immune checkpoint inhibitors and targeted therapies (e.g., FGFR2 and HER2 inhibitors) have demonstrated clinical benefit in advanced disease, suggesting a role for personalized systemic therapy [14,15,16]. Despite these advances, clinical consensus on optimal surgical approaches (e.g., wedge resection vs. segment IVb + V resection), the extent of lymphadenectomy, and the real-world benefit of adjuvant therapies remains limited.

This multicenter retrospective study aimed to evaluate the clinicopathological predictors of survival and recurrence in patients that underwent curative-intent resection for GBC. We also explored the impact of the surgical approach, postoperative adjuvant chemotherapy, and year of treatment on the patient outcomes, with the goal of informing individualized treatment strategies.

## 2. Materials and Methods

### 2.1. Patient Selection

**Figure 1:**
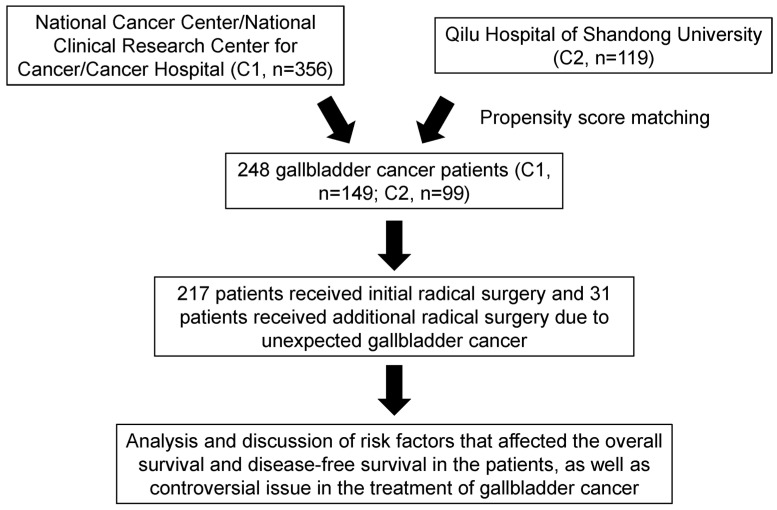
Flowchart of patient enrollment in the study. Abbreviations: C1 or C2: Center 1 or Center 2.

A retrospective analysis was conducted on data from patients who underwent resection for primary GBC between January 2010 and January 2022 at two Chinese medical centers: the National Cancer Center/National Clinical Research Center for Cancer/Cancer Hospital of Chinese Academy of Medical Sciences and Peking Union Medical College and the Qilu Hospital of Shandong University. The flowchart of the study is shown in Figure 1. Detailed information on the patients’ demographic characteristics, perioperative data, adjuvant chemotherapy, and oncological outcomes was obtained from electronic medical records and is presented in Table 1. This study was reviewed and approved by the Institutional Review Boards of both medical centers and was conducted in accordance with the Declaration of Helsinki [17].

### 2.2. Inclusion and Exclusion Criteria

The inclusion criteria were as follows: (1) patients diagnosed with primary GBC without concomitant malignancies; (2) patients who underwent successful resection of the primary tumor at our institution, or those who underwent a cholecystectomy for benign indications but were incidentally diagnosed with GBC of T1b stage or higher on preoperative or prior pathology and required subsequent surgery; (3) patients with R0 resection confirmed by postoperative pathology.

The exclusion criteria were as follows: (1) patients diagnosed with non-GBC or concurrent malignancies; (2) patients with significant missing data on clinically relevant outcome measures; (3) patients lost to follow-up without undergoing the first postoperative review at our institution or perioperative deaths.

### 2.3. Diagnosis and Treatment Methods

The initial diagnostic and treatment decisions for patients with GBC are formulated by a multidisciplinary team (MDT) consisting of hepatobiliary surgeons, medical oncologists, pathologists, and radiologists after consultation.

Preoperative radiological examinations include contrast-enhanced CT and MRI of the chest, abdomen, and pelvis to systematically assess the resectability of the primary site. The extent of surgical resection is determined based on preoperative radiological examinations and intraoperative findings. When preoperative radiological examinations suggest the possibility of lymph node metastasis or intraoperative frozen section pathology analysis indicates lymph node metastasis or the inability to achieve negative margins at the cystic duct stump, extrahepatic bile duct resection may be performed. In cases where the tumor involves the hepatic hilum, especially the right hepatic artery, an extended hemi-hepatectomy may be considered. Pathological examination results of GBC tissue include the TNM staging system jointly issued by the American Joint Committee on Cancer (AJCC, 8th Edition), lymph node status, and histological grade.

Regional lymph node dissection for GBC is categorized into three stations. The first station includes lymph nodes along the cystic duct and peri-choledochal nodes (No. 12b). The second station comprises lymph nodes posterior to the portal vein (No. 12p), along the proper hepatic artery (No. 12a), adjacent to the common hepatic artery (No. 8), and superior to the pancreatic head (No. 13a). The third station involves distant lymph nodes, including those around the celiac trunk (No. 9); nodes around the pancreatic head (No. 13b, No. 17, and No. 18); nodes around the superior mesenteric artery (No. 14); and para-aortic lymph nodes (No. 16).

For incidental T1b GBC, the recommended supplementary surgery consists of a wedge resection (a wedge resection is defined as the removal of a 2 cm margin of liver tissue surrounding the gallbladder bed) of the liver combined with regional lymph node dissection. For T2a-stage disease, wedge resection combined with regional lymph node dissection is performed. The standard surgical procedure for T2b-stage GBC is an extended cholecystectomy, which includes either a wedge resection of the gallbladder bed or a segment IVb + V hepatectomy, combined with regional lymph node dissection. For stages T3 and above, a segment IVb + V hepatectomy with regional lymph node dissection is recommended. Due to surgical requirements, lymph node dissection in some patients involves the simultaneous removal of both regional and distant lymph nodes.

Postoperative infections include surgical site abscess, sepsis, and fever without microbiological evidence (defined as a temperature > 38 °C persisting for more than 3 days, occurring after postoperative day 3). POAC is defined as any oral or intravenous chemotherapy—either monotherapy (e.g., oral capecitabine) or combination therapy (e.g., S-1 combined with oxaliplatin)—initiated within 3 months following surgery.

### 2.4. Examination Indicators

In addition to preoperative routine blood tests and selected biochemical tests, the body mass index (BMI) was calculated as weight (kg) divided by height squared (m^2^). The prognostic nutritional index (PNI) was calculated using the following formula: PNI = (lymphocyte count (10^9^/L) × 5) + (albumin (g/L)). The American Society of Anesthesiologists (ASA) score was categorized from ASA1 to ASA4. The neutrophil–lymphocyte ratio (NLR), lymphocyte–monocyte ratio (LMR), and platelet–lymphocyte ratio (PLR) were derived from preoperative routine blood test parameters. The CA19-9 plus NLR (CAN) score was defined as follows: low score (CA19-9 < 30 and NLR < 3.5), moderate score (CA19-9 < 30 or NLR < 3.5), and high score (CA19-9 > 30 and NLR > 3.5).

### 2.5. Follow-Up

The patients underwent their first postoperative review the first month after the surgical resection, followed by tumor marker testing; contrast-enhanced CT or other imaging examinations; medical history inquiry; and a physical examination every three months for two years postoperatively, then every six months thereafter. The patients who underwent curative surgery for GBC required follow-up visits and treatment according to the treatment cycle. All data were reviewed via the electronic medical record system and integrated by two researchers independently to obtain the overall survival (OS) and disease-free survival (DFS) of patients, with follow-up until 30 July 2023. The patients who did not return for follow-up visits at our institution had their recurrence status and survival information confirmed by telephone by research personnel.

### 2.6. Statistical Analysis

Statistical analysis was performed using SPSS version 24.0 software and R statistical software (version 4.2.0). Continuous data are presented as the mean ± standard deviation for normally distributed variables, and as median [interquartile range (Q1, Q4)] for non-normally distributed variables. Categorical data were calculated using both frequencies and percentages. Univariate and multivariate Cox regression models were used to analyze prognostic factors. Variables with *p* < 0.05 in the univariate analysis were selected for the multivariate Cox regression analysis. The results are presented as hazard ratios (HRs) and 95% confidence intervals (CIs). Multiple imputation (using the mice package) was employed to impute missing values, with the parameter set to m = 8. The imputation was based on imp values, which involved selecting the data with the best imputation effect for the downstream analysis (Figures S1 and S2). Kaplan–Meier methods were employed for the survival analysis, and the log-rank test was used to compare the clinically significant subgroup indicators. In this observational study, propensity score matching (PSM) was used to achieve a balanced covariate distribution between the two treatment groups (Tables S1 and S2). The best cut-off values for the inflammatory biomarkers were determined by the receiver operating characteristic (ROC) curve (Table S3). All reported *p* values are two-tailed, with a significance level set at 0.05.

## 3. Results

### 3.1. Patients’ Baseline Characteristics

The median follow-up time was 44.7 (range, 0.3–159.3) months. Among the 248 patients, 31 underwent supplementary surgery for incidental GBC (partial hepatectomy + hepatoduodenal lymph node dissection) and 217 patients underwent initial curative surgery. Of these, 25 cases underwent laparoscopic surgery (with 4 cases converted to open surgery), and 227 cases underwent open surgery. Among those who underwent curative surgery, 217 underwent concomitant partial hepatectomy (including 7 cases of extended right hepatectomy, 2 cases of hepatopancreaticoduodenectomy, 9 cases of extrahepatic bile duct resection + internal drainage, 13 cases of combined right hemicolectomy or partial gastrectomy, 3 cases of combined hysterectomy and unilateral or bilateral salpingo-oophorectomy, and 3 cases of combined omentectomy or diaphragm resection, while the remaining cases underwent segment IVb + V hepatectomy). Among the patients who underwent curative surgery, 67 experienced postoperative complications (requiring intervention for Clavien–Dindo grade II or higher complications in 55 cases). Postoperative pathological examination revealed 219 cases of adenocarcinoma, 12 cases of adenosquamous carcinoma, 5 cases of papillary carcinoma, 12 cases of neuroendocrine carcinoma, and other pathological types. A total of 106 patients received postoperative adjuvant chemotherapy (POAC) (Table 1).

**Figure 2:**
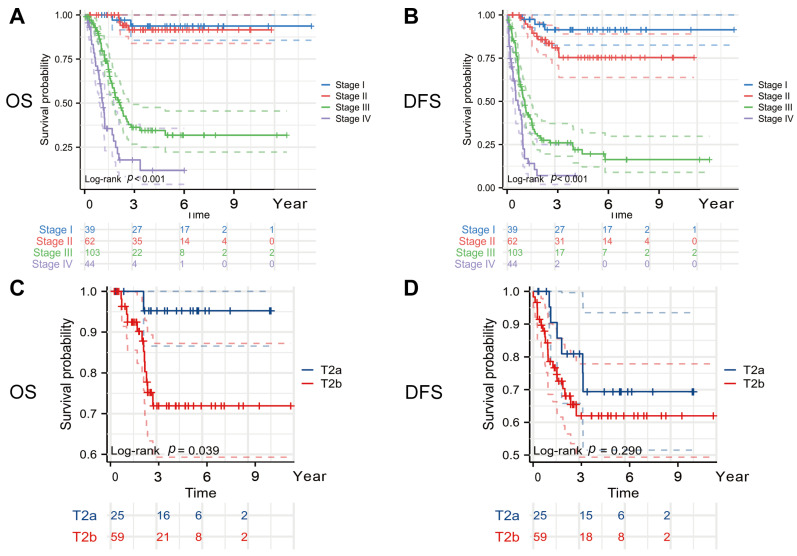
Kaplan–Meier curves. (**A**) OS curves according to TNM stage, (**B**) DFS curves according to TNM stage, (**C**) OS curves according to T stage, and (**D**) DFS curves according to T stage. Abbreviations: OS, overall survival; DFS, disease-free survival. All reported *p* values are two-tailed, with a significance level set at 0.05.

There was a total of 124 recurrences. Ninety patients died from malignant tumors of the gallbladder and associated complications during follow-up. The cumulative survival rates at 1, 3, and 5 years postoperatively were 83.6% (95% CI 78.9–88.5), 65.8% (95% CI 59.7–72.5), and 57.4% (95% CI 50.8–64.8), respectively. Across the entire cohort, there was a significant difference in the OS between the early and late stages of the disease (stage I vs. stage IV: HR = 28.574, 95% CI 13.958–58.213, *p* < 0.001). The median survival time for the stage III patients treated with surgery was 24.8 months (95% CI 19.5–32.6), while for the stage IV patients, the median OS was 12.9 months (95% CI 10.7–20.3). The median DFS time for the stage III patients was 12.3 months (95% CI 9.4–17.1), and for the stage IV patients, the median survival time was 7.4 months (95% CI 4.0–10.8) (Figure 2A,B).

### 3.2. Prognostic Factors in the Entire Cohort

The results of univariate and multivariate analyses of factors that influenced the prognosis of GBC in the entire cohort are detailed in Table 2 and Table 3. Univariate analysis results showed that elevated preoperative CA19-9 (>30 U/mL); NLR (>3.5); CA19-9 plus NLR (CAN) score; surgical approach (supplementary surgery vs. curative surgery); serosal invasion or worse; poor/moderate-to-poor tumor differentiation; tumor invasion of the liver, nerves, or vessels; T3–T4 stage; N1–N2 stage; M1 stage; postoperative complications; and postoperative infection were risk factors for the OS of GBC patients (*p* < 0.05). These indicators were also risk factors for the DFS (*p* < 0.05, Table 2). The multivariate analysis results showed that an elevated preoperative CA19-9 (>30 U/mL), poor/moderate-to-poor tumor differentiation, T3–T4 stage, N1–N2 stage, and M1 stage were independent risk factors for the OS of GBC (*p* < 0.05). Poor/moderate-to-poor tumor differentiation, T3–T4 stage, N1–N2 stage, and M1 stage were independent risk factors for the DFS of GBC (*p* < 0.05, Table 3). A high CAN score was the only inflammatory biomarker related to both the OS and DFS (*p* < 0.05).

### 3.3. Subgroup Analysis

Studies have shown that tumors in the T2a and T2b stages, due to their different locations, may have distinct survival outcomes [7,18,19]. Tumors in the T2b stage, being closer to the gallbladder bed, are more prone to spread through veins and lymphatic vessels, leading to a poorer prognosis. We found that the T2a-stage patients had a better OS (5-year OS rate, T2a vs. T2b: 95.2% vs. 71.9%) (HR = 6.417, 95% CI = 2.058–20.084, log-rank *p* = 0.039) and a longer DFS (5-year DFS rate, T2a vs. T2b: 69.8% vs. 62.1%) (HR = 1.634, 95% CI = 0.713–3.737, log-rank *p* = 0.290), although the difference in the DFS between the two groups was not statistically significant (Figure 2C,D).

**Figure 3:**
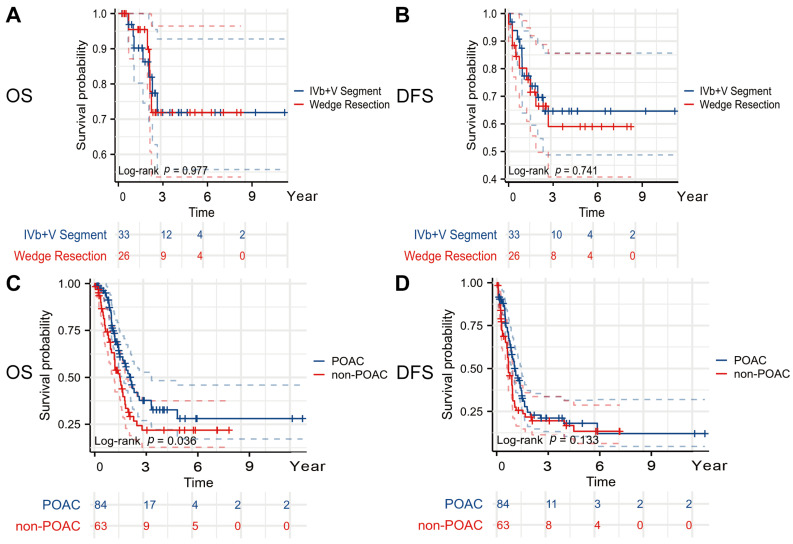
Kaplan–Meier curve in subgroup analyses. (**A**) OS curve according to extent of liver resection (IVb + V segment vs. wedge resection), (**B**) DFS curve according to extent of liver resection (IVb + V segment vs. wedge resection), (**C**) OS curve according to POCA in patients with stages III–IV, and (**D**) DFS curve according to POCA in patients with stages III–IV. Abbreviations: OS, overall survival; DFS, disease-free survival; POAC, postoperative adjuvant chemotherapy. All reported *p* values are two-tailed, with a significance level set at 0.05.

There is still no definitive conclusion or evidence on whether hepatic resection should be combined for the T1b and T2a stages. As for patients with GBC at the T2b stage, it also remains unclear whether wedge resection or segment IVb + V hepatectomy offers superior outcomes. Among the patients with T2b-stage GBC, those who underwent a segment IVb + V hepatectomy had an OS (HR = 0.986; 95% CI: 0.314–3.090; log-rank *p* = 0.977) and DFS (HR = 1.165; 95% CI: 0.470–2.884; log-rank *p* = 0.741) comparable with those who underwent a wedge resection, with no statistically significant differences observed between the two surgical approaches (Figure 3A,B).

In the patients with stages III–IV, those who underwent POAC had a longer OS (24.8 months vs. 17.3 months, HR = 1.575, 95% CI 1.010–2.447, *p* = 0.036) and DFS (12.0 months vs. 8.0 months, HR = 1.285, 95% CI 0.877–1.859, *p* = 0.133) compared with those who did not (Figure 3C,D).

The patients who underwent surgical treatment for GBC between 2017 and 2022 had a longer OS (HR = 0.593, 95% CI = 0.373–0.943, *p* = 0.024) and DFS (HR = 0.874, 95% CI 0.754–1.248, *p* = 0.334) than those treated between 2010 and 2016 (Figure S3).

## 4. Discussion

This retrospective study analyzed the treatment and prognosis of GBC patients who underwent radical surgery at two included centers over a period of 12 years. We attempted to identify the independent risk factors that affected the survival and tumor recurrence of the GBC patients. The results indicate that an elevated preoperative CA19-9 (>30 U/mL), high CAN score, poor/moderate-to-poor tumor differentiation, T3–T4 stage, N1–N2 stage, and M1 stage were independent risk factors that affected the OS of the GBC patients (*p* < 0.05). Moreover, a high CAN score, poor/moderate-to-poor tumor differentiation, T3–T4 stage, N1–N2 stage, and M1 stage were independent risk factors that affected the DFS of the GBC patients (*p* < 0.05). Specifically, a high CAN score emerged as the only inflammatory biomarker independently associated with both the OS and DFS, providing a potentially useful and easily accessible prognostic tool for clinicians.

For patients with stage IV GBC, a large-scale study from Japan suggests that radical surgery does not improve the prognosis [20]. However, Chen et al. found that compared with a median survival time of only 2.3 months for patients with advanced GBC who did not undergo surgery, those who underwent radical surgery experienced extended survival [21,22]. In our study, the median survival time for the stage IV GBC patients who underwent radical surgery reached 12.9 months (95% CI 10.7–20.3 months). This could be attributed to more standardized comprehensive treatment protocols. We conducted a subgroup analysis of the stage III–IV GBC patients, which revealed that among the 147 patients who underwent postoperative adjuvant treatment, their survival period was prolonged (*p* = 0.036), although the tumor recurrence time did not significantly differ from the untreated group (*p* = 0.133). It should be noted that the time span of this study was relatively long. The guideline-recommended postoperative adjuvant chemotherapy regimen using capecitabine was introduced in 2017. In addition, immune checkpoint inhibitors have been increasingly explored in recent years as potential adjuvant treatments for GBC. Consequently, the postoperative adjuvant treatment regimens included in this study are diverse and evolving, making detailed classification and analysis unfeasible within the current scope. In future research, we plan to expand the sample size and conduct a more comprehensive classification and evaluation of postoperative adjuvant therapies.

Similar to the findings reported by Kuipers et al., as the tumor stage increased, the extent of liver resection also expanded accordingly, sometimes inevitably involving co-resection of the stomach, colon, duodenum, and pancreas [8]. This resulted in a higher postoperative complication rate (41.2%) and postoperative infection rate (32.4%) for advanced GBC patients. In our study, postoperative infection emerged as a dual-risk factor for GBC patient survival and recurrence. Therefore, the cautious selection of surgical methods to minimize postoperative complications in advanced GBC patients remains crucial. Our study’s analysis of the prognosis of GBC patients over time indirectly confirms this point. The results show that patients who underwent radical surgery after 2017 had longer survival periods, possibly benefiting from advancements in surgical techniques and perioperative management, as well as the discovery and application of targeted drugs and immune checkpoint inhibitors [14,15,23,24,25]. Moreover, with the improvement in disease screening and increased public health awareness, more patients at an early stage of the disease underwent surgical treatment, contributing to a better prognosis. Further in-depth research is needed in the future.

GBC on the liver side, lacking a serosal layer and adjacent to the liver, is prone to liver metastasis once lymph node metastasis occurs, especially intrahepatic metastasis [26]. Although our study identified lymph node metastasis, perineural invasion, and vascular invasion as independent risk factors significantly influencing patient recurrence and prognosis, we did not observe a definitive correlation between the extent of lymphadenectomy and patient survival. Nevertheless, it is important to emphasize that the number of lymph nodes removed is crucial for accurately evaluating lymph node metastasis. It is generally recommended that at least six lymph nodes be resected during a gallbladder lymphadenectomy.

Although this study found that the tumor infiltration depth (T stage) and lymph node metastasis (N stage) were independent risk factors that affected the disease recurrence and prognosis, no definite correlation was found between the extent of lymph node dissection and patient survival. In this study, 163 patients underwent lymph node dissection, where 83 cases involved the dissection of stations 8 and 12 (No. 8 + No. 12); 82 cases involved the dissection of stations 8, 12, and 13 (No. 8 + No. 12 + No. 13); and 21 cases involved dissection of four or more stations (No. 8 + No. 12 + No. 13 + others). However, the number of resected lymph nodes is important for accurately assessing lymph node metastasis [11,27,28,29]. It is generally believed that lymph node dissection for GBC should involve the resection of at least six or more lymph nodes [30]. Some studies suggest that in T2a-stage GBC located on the serosal side, drainage occurs through one to two gallbladder veins into the porta hepatis or nearby liver parenchyma, while in T2b-stage GBC, multiple short gallbladder veins directly connect to the intrahepatic portal vein, promoting tumor cell spread. T2b-stage GBC is more likely to have micro-metastases in the liver due to richer venous and lymphatic drainage leading into the liver parenchyma [13]. We also found that the T2a-stage patients had a better OS (*p* = 0.039) and longer DFS (*p* = 0.290). Therefore, some studies suggest that for T2b-stage GBC, standard resection of segments IVb and V of the liver is more effective than wedge resection of the liver 2 cm away from the tumor [31]. However, the subgroup analysis results in this study did not show a difference in the prognosis and recurrence between the two surgical methods, although the patients that underwent IVb + V segment resection had slightly better DFS data. It should be noted that unless the liver and gallbladder are resected together, pathologists are likely to misinterpret the location of the tumor, especially in the pathological specimens of incidental GBC patients in stage T2. In addition, due to differences in surgical expertise and cognition between different surgeons, wedge resection performed by one expert may be considered as IVb + V segment liver resection by another expert. Therefore, the results of this study still need to be interpreted with caution.

In fact, modern cancer prognostic assessment is no longer confined to traditional clinicopathological features but encompasses both molecular and systemic factors [32]. In gallbladder cancer, recurrent genetic alterations, particularly KRAS and TP53 mutations, are associated with more aggressive tumor behavior and poorer survival outcomes, underscoring their utility as prognostic biomarkers [33]. Moreover, the expression of immune checkpoint proteins (such as PD-L1) not only provides prognostic information but also identifies patients who may benefit from PD-1/PD-L1-targeted therapies [34]. Although there have been no recent advances in adjuvant therapy for GBC, in the context of comprehensive treatment for advanced disease, recent clinical trials (for example, TOPAZ-1) have shown that adding a PD-L1 inhibitor can significantly prolong the overall survival [14,35]. Other emerging targeted agents against FGFR, IDH1/2, and HER2 alterations further highlight the promise of precision oncology in GBC treatment [16,36]. Going forward, incorporating discussions of genetic biomarkers and systemic therapies into our analyses may yield fresh insights for individualized patient cure.

In summary, this study retrospectively reviewed the treatment patterns of GBC patients who underwent curative-intent surgery over a 12-year period at two major institutions: the Cancer Hospital of the National Cancer Center and the Qilu Hospital of Shandong University. Through comprehensive analyses, we identified several independent risk factors associated with overall survival and tumor recurrence in GBC patients. In addition, we sought to address certain clinically relevant but unresolved questions regarding surgical strategy and adjuvant therapy. However, it is important to acknowledge several limitations. Gallbladder cancer is a relatively rare malignancy, and the retrospective nature of the study, along with its extended time span, may have introduced unavoidable bias. Moreover, treatment modalities and diagnostic criteria have evolved over the years, potentially affecting the consistency of the data. Therefore, our findings should be interpreted with caution, and further validation is needed. Future studies with larger patient cohorts and long-term prospective follow-up are warranted to provide more definitive evidence and to inform standardized treatment guidelines for gallbladder cancer. 

## 5. Conclusions

This retrospective study confirmed that radical surgery remains a key treatment for GBC, with survival influenced by the tumor stage, differentiation, metastasis, and postoperative infection. Stage IV patients may benefit from surgery, especially when combined with adjuvant therapy. While extended lymphadenectomy and segment IVb + V resection are often recommended, their survival advantage remains uncertain. Advances in surgical techniques and systemic therapies have improved outcomes in recent years. Further large-scale, prospective studies are needed to refine the treatment strategies for GBC.

## Figures and Tables

**Table 1:** Baseline characteristics of GBC patients.

Characteristics	Overall (n = 248)	Characteristics	Overall (n = 248)
**Gender**		**Tumor location**	
Female	153 (61.7%)	Gallbladder body	94 (44.1%)
Male	95 (38.3%)	Gallbladder fundus	81 (38%)
**BMI**	24.3 (22.2, 26.7)	Gallbladder neck	22 (10.3%)
**Biliary disease**		Cystic duct	16 (7.5%)
Gallstone	72 (79.1%)	Beyond recognition	35 (14.1%)
Gallbladder polyps	12 (13.2%)	**Tumor infiltration depth**	
Gallbladder adenomyomatosis	6 (6.6%)	Mucosal layer	26 (10.5%)
Other	1 (1.1%)	Muscularis propria	49 (19.8%)
No biliary disease	157 (63.3%)	Serosal layer	114 (46%)
**Metabolic syndrome**		Extraserous	59 (23.8%)
Yes	38 (15.3%)	**Tumor differentiation**	
No	210 (84.7%)	Well	39 (15.7%)
**CA19-9 (U/mL)**	19.7 (10.3, 101.2)	Moderately	89 (35.9%)
**PNI**	57.8 (21.4, 84.1)	Moderately-to-poorly	17 (6.9%)
**NLR**	3.4 (1.4, 10.8)	Poorly	103 (41.5%)
**ASA grade**		**LMR**	3.4 (1.7, 7.9)
I	7 (2.8%)	**PLR**	119 (28.7, 204.8)
II	234 (94.4%)	**Neural invasion**	
III	7 (2.8%)	No	176 (71%)
**Type of operation**		Yes	72 (29%)
Radical surgery	217 (87.5%)	**Vascular invasion**	
Supplementary operation	31 (12.5%)	No	180 (72.6%)
**Liver metastasis**		Yes	68 (27.4%)
No	204 (82.3%)	**T stage**	
Yes	44 (17.7%)	T1	39 (15.7%)
**Extent of liver resection**		T2	97 (39.1%)
Wedge resection (2 cm)	86 (40.6%)	T3	101 (40.7%)
IVb + V segment resection	119 (56.1%)	T4	11 (4.4%)
IV V and VIII segment resection	5 (2.4%)	**N stage**	
Right hemihepatectomy	2 (0.9%)	Nx ^1^	29 (11.7%)
**Lymph node dissection**		N0	131 (52.8%)
No	29 (11.7%)	N1	66 (26.6%)
Yes	219 (88.3%)	N2	22 (8.9%)
**Lymph node dissection area**		**M stage**	
NA	29 (11.7%)	M0	226 (91.1%)
No. 8 + No. 12	117 (47.0%)	M1	22 (8.9%)
No. 8 + No. 12 + No. 13	82 (32.8%)	**Postoperative adjuvant chemotherapy**	
No. 8 + No. 12 + No. 13 + others	21 (8.5%)	No	142 (57.3%)
**Postoperative infection**		Yes	106 (42.7%)
No	205 (82.7%)	**Complications**	
Yes	43 (17.3%)	No	181 (73%)
**Tumor histological type**		Yes	67 (27%)
Adenocarcinoma	219 (88.3%)	**Length of hospital stay**	14 (11.75, 19)
Others	29 (11.7%)	**Preoperative hospital stays**	6 (4, 8)

^1^ Nx, for patients classified as stage T1a, only a cholecystectomy was performed without lymph node dissection. Abbreviations: GBC, gallbladder cancer.

**Table 2:** Univariate Cox analysis of demographics and clinicopathologic characteristics for OS and DFS of GBC patients.

Characteristics	Total	OS		DFS	
	(N)	HR (95% CI)	*p* Value	HR (95% CI)	*p* Value
**Age (year)**	248				
≤60	107	Reference		Reference	
>60	141	1.382 (0.899–2.123)	0.140	1.301 (0.904–1.873)	0.157
**Gender**	248				
Female	153	Reference		Reference	
Male	95	1.259 (0.828–1.913)	0.281	1.154 (0.804–1.655)	0.438
**BMI**	248				
≤28	204	Reference		Reference	
>28	44	0.960 (0.559–1.648)	0.881	0.697 (0.418–1.164)	0.168
**Metabolic syndrome**	248				
Yes	38	Reference		Reference	
No	210	0.949 (0.545–1.653)	0.855	1.025 (0.629–1.672)	0.921
**CA19-9 (U/mL)**	248				
≤30	144	Reference		Reference	
>30	104	3.557 (2.305–5.490)	**<0.001**	2.915 (2.029–4.188)	**<0.001**
**PNI**	248				
≤50	104	Reference		Reference	
>50	144	0.945 (0.546–1.546)	0.878	1.120 (0.679–1.610)	0.944
**NLR**	248				
≤3.5	94	Reference		Reference	
>3.5	154	1.875 (1.247–2.743)	**0.024**	2.018 (1.378–2.781)	**0.007**
**LMR**	248				
≤3.0	134	Reference		Reference	
>3.0	114	0.984 (0.648–1.457)	0.754	1.246 (0.724–1.647)	0.781
**PLR**	248				
≤100	151	Reference		Reference	
>100	97	0.979 (0.546–1.456)	0.681	1.123 (0.549–1.782)	0.841
**CAN**	248				
Low/moderate score	176	Reference		Reference	
High score	72	2.781 (1.841–4.016)	**<0.001**	2.548 (1.453–3.412)	**<0.001**
**Type of operation**	248				
Radical surgery	217	Reference		Reference	
Supplementary operation	31	0.339 (0.138–0.836)	**0.019**	0.469 (0.238–0.925)	**0.029**
**Liver metastasis**	248				
No	204	Reference		Reference	
Yes	44	2.834 (1.782–4.509)	**<0.001**	2.761 (1.861–4.096)	**<0.001**
**Tumor infiltration depth**	248				
Intramucosal	75	Reference		Reference	
Extramucosal	173	3.825 (2.119–6.903)	**<0.001**	3.694 (2.259–6.041)	**<0.001**
**Tumor differentiation**	248				
Poor/moderate to poor	120	Reference		Reference	
Good/moderate	128	2.990 (1.936–4.617)	**<0.001**	3.025 (2.088–4.382)	**<0.001**
**Tumor histological type**	248				
Adenocarcinoma	219	Reference		Reference	
Others ^1^	29	0.914 (0.582–1.594)	0.751	1.072 (0.689–1.710)	0.843
**Neural invasion**	248				
No	176	Reference		Reference	
Yes	72	2.263 (1.468–3.489)	**<0.001**	2.935 (2.037–4.229)	**<0.001**
**Vascular invasion**	248				
No	180	Reference		Reference	
Yes	68	2.712 (1.756–4.189)	**<0.001**	3.013 (2.072–4.382)	**<0.001**
**T**	248				
T1 and T2	136	Reference		Reference	
T3 and T4	112	6.635 (4.051–10.867)	**<0.001**	5.123 (3.462–7.581)	**<0.001**
**N**	248				
N0 ^2^	160	Reference		Reference	
N1 and N2	88	5.005 (3.255–7.694)	**<0.001**	4.693 (3.237–6.805)	**<0.001**
**M**	248				
M0	226	Reference		Reference	
M1	22	5.104 (2.933–8.879)	**<0.001**	5.571 (3.308–9.382)	**<0.001**
**Complications**	248				
No	181	Reference		Reference	
Yes	67	2.372 (1.557–3.613)	**<0.001**	2.190 (1.519–3.157)	**<0.001**
**Postoperative infection**	248				
No	205	Reference		Reference	
Yes	43	3.371 (2.148–5.288)	**<0.001**	2.882 (1.931–4.302)	**<0.001**

^1^ Adenosquamous carcinoma, papillary carcinoma, and neuroendocrine carcinoma. ^2^ Given that the lymph node metastasis rate in T1a disease was below 1%, we conservatively grouped these Nx cases together with N0 for all subsequent analyses. Abbreviations: OS, overall survival; DFS, disease-free survival; HR, hazard ratio; CI, confidence interval; GBC, gallbladder cancer; CI, confidence interval; NLR, neutrophil-to-lymphocyte ratio; PLR, platelet-to-lymphocyte ratio; PNI, prognostic nutritional index; LMR: lymphocyte-to-monocyte ratio. The bold *p* value indicates statistically significant.

**Table 3:** Multivariate Cox analysis of demographics and clinicopathologic characteristics for OS and DFS of GBC patients.

Characteristics	Total	OS		DFS	
	(N)	Hazard Ratio (95% CI)	*p* Value	Hazard Ratio (95% CI)	*p* Value
**CA19-9 (U/mL)**	248				
≤30	144	Reference		Reference	
>30	104	1.709 (1.033–2.828)	**0.037**	1.248 (0.786–1.248)	0.154
**NLR**	248				
≤3.5	94	Reference		Reference	
>3.5	154	1.878 (0.936–2.579)	0.064	1.456 (0.879–1.424)	0.078
**CAN**					
Low/moderate score	176	Reference		Reference	
High score	72	1.875 (1.3474–2.478)	**0.009**	1.713 (1.348–2.154)	**0.034**
**Liver metastasis**	248				
No	204	Reference		Reference	
Yes	44	1.050 (0.630–1.752)	0.851	0.920 (0.589–1.436)	0.713
**Tumor infiltration depth**	248				
Intramucosal	75	Reference		Reference	
Extramucosal	173	1.046 (0.532–2.055)	0.896	1.308 (0.747–2.291)	0.348
**Tumor differentiation**	248				
Poor/moderate to poor	120	Reference		Reference	
Good/moderate	128	1.636 (1.024–2.615)	**0.040**	1.728 (1.163–2.568)	**0.007**
**Neural invasion**	248				
No	176	Reference		Reference	
Yes	72	0.872 (0.540–1.408)	0.575	1.295 (0.858–1.954)	0.219
**Vascular invasion**	248				
No	180	Reference		Reference	
Yes	68	1.254 (0.765–2.056)	0.370	1.154 (0.743–1.793)	0.524
**T**	248				
T1 and T2	136	Reference		Reference	
T3 and T4	112	3.129 (1.758–5.569)	**<0.001**	2.397 (1.499–3.833)	**<0.001**
**N**	248				
N0 ^1^	160	Reference		Reference	
N1 and N2	88	3.039 (1.853–4.985)	**<0.001**	2.506 (1.618–3.882)	**<0.001**
**M**	248				
M0	226	Reference		Reference	
M1	22	3.235 (1.822–5.743)	**<0.001**	3.085 (1.770–5.376)	**<0.001**
**Complications**	248				
No	181	Reference		Reference	
Yes	67	0.855 (0.423–1.728)	0.663	1.114 (0.624–1.988)	0.715
**Postoperative infection**	248				
No	205	Reference		Reference	
Yes	43	1.403 (0.929–2.120)	0.108	1.712 (0.871–3.366)	0.119

^1^ Given that the lymph node metastasis rate in T1a disease was below 1%, we conservatively grouped these Nx cases together with N0 for all subsequent analyses. Abbreviations: OS, overall survival; DFS, disease-free survival; HR, hazard ratio; CI, confidence interval; GBC, gallbladder cancer. The bold *p* value indicates statistically significant.

## Data Availability

The data presented in this study is available upon request from the corresponding author.

## Supplementary Materials

The following supporting information can be downloaded from https://www.mdpi.com/article/10.3390/curroncol32060328/s1: Figure S1: Visualization of multiple imputation results. (A) Missing data pattern plot, (B) percentage of missing cases and missing data pattern plot, and (C) summary of multiple imputation values. Figure S2: Multiple imputation values of the multiple imputation results. Figure S3: Kaplan–Meier curve according to the operative year. (A) OS curve according to the operative year and (B) DFS curve according to the operative year. Table S1: Baseline characteristics of GBC patients before PSM analysis. Table S2: Baseline characteristics of GBC patients after PSM analysis. Table S3: Results of ROC analysis for inflammatory biomarkers.
