# Supplementary material for: Prognostic Factors and Treatment Outcomes in Gallbladder Cancer Patients Undergoing Curative Surgery: A Multicenter Retrospective Cohort Study
Source: Curr Oncol. 2025 Jun 3;32(6):328. doi: 10.3390/curroncol32060328 (PMC12192010; doi:10.3390/curroncol32060328)
Supplement: Supplementary file 1 [file curroncol-32-00328-s001.zip › curroncol-3638712-supplementary.pdf]

Supplementary Materials

1. Supplementary Figures

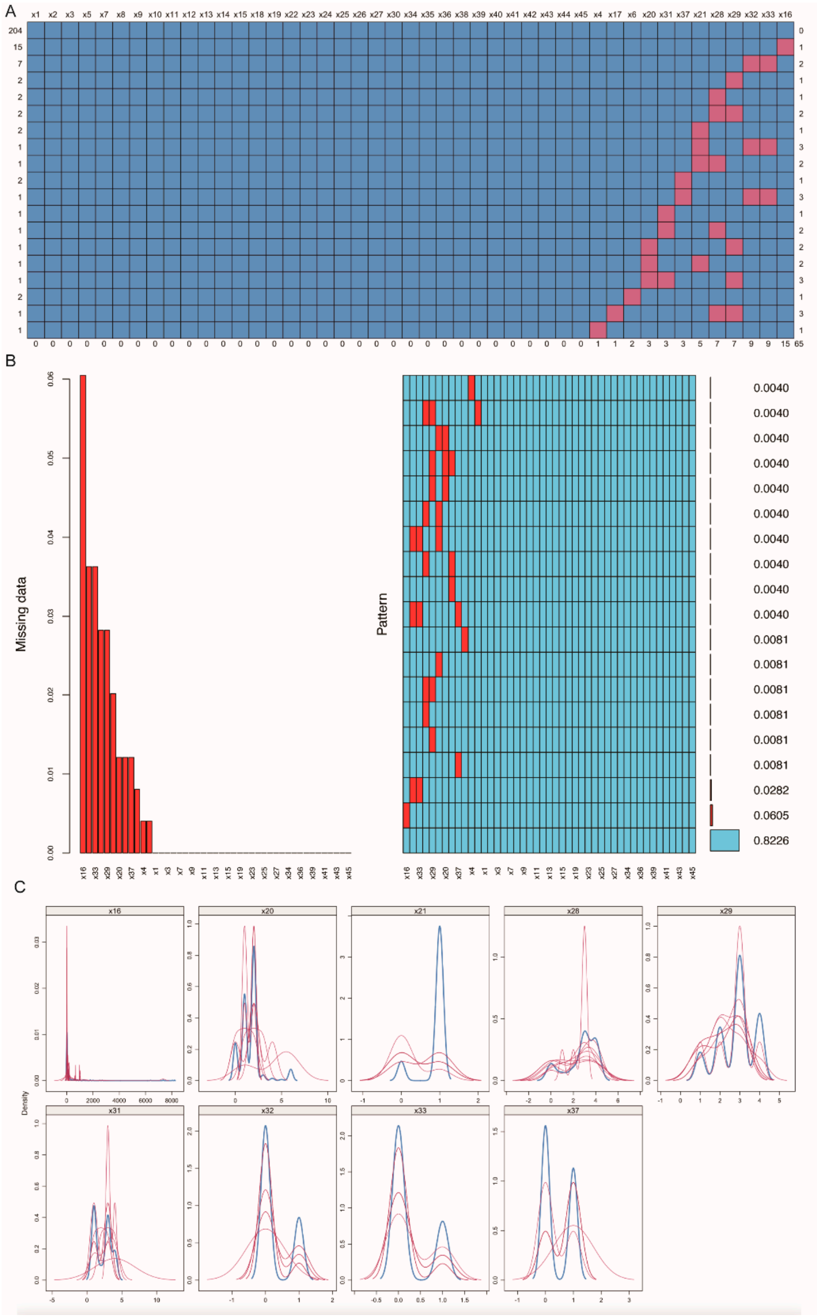

Figure S1. Visualization of multiple imputation results. (**A**) Missing data pattern plot, (**B**) Percentage of missing cases and missing data pattern plot, (**C**) Summary of multiple imputation values.

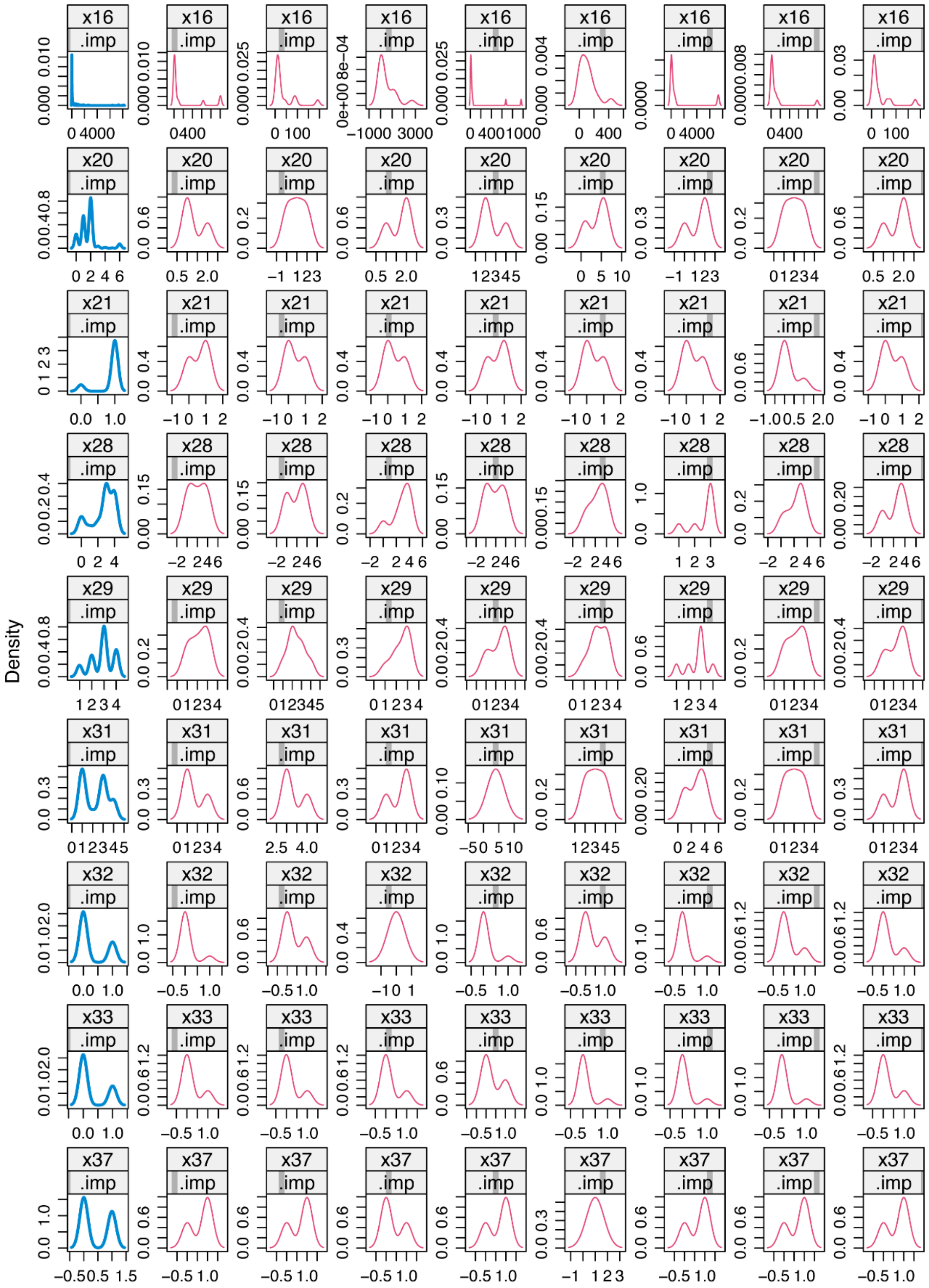

Figure S2. Multiple imputation values of the multiple imputation results.

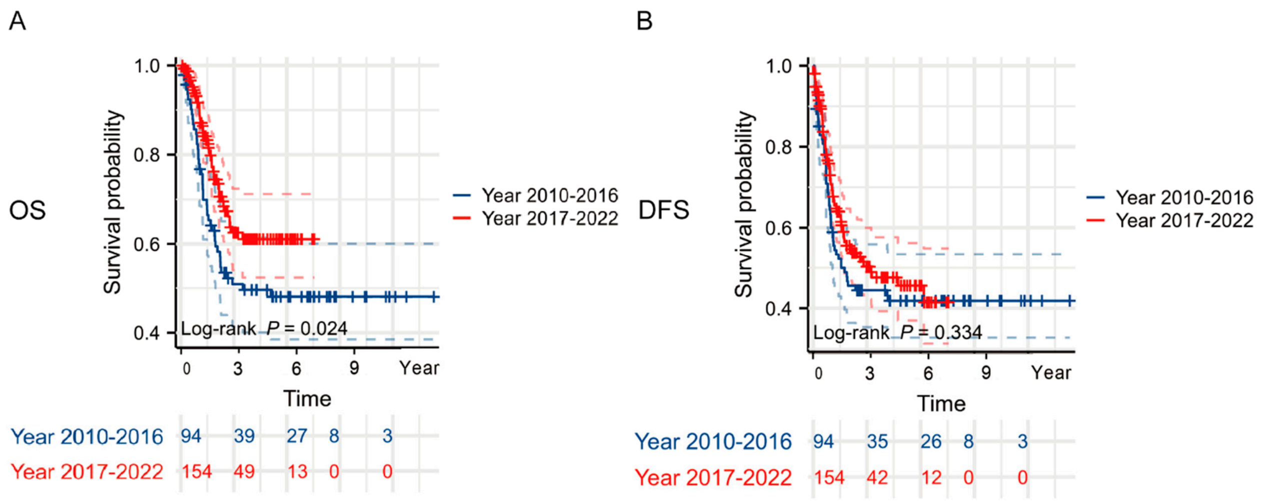

Figure S3. Kaplan–Meier curve according to the operative year. (**A**) OS curve according to the operative year, (**B**) DFS curve according to the operative year. Abbreviations: OS, overall survival; DFS, disease-free survival.

2. Supplementary Tables

Table S1. Baseline characteristics of GBC patients before PSM analysis.

Characteristics		Center 1	Center 2	*P*
		**356**	**119**	
**Age (Year)**	> 60	216 (60.7%)	65 (54.6%)	0.359
	≤ 60	140 (39.3%)	54 (45.4%)	
**Gender**	Female	210 (59.0%)	77 (64.7%)	0.384
	Male	146 (41.0%)	42 (35.3%)	
**BMI**	> 28	48 (13.5%)	24 (20.2%)	0.170
	≤ 28	308 (86.5%)	95 (79.8%)	
**Metabolic syndrome**	No	292 (82.0%)	102 (85.7%)	0.496
	Yes	64 (18.0%)	17 (14.3%)	
**CA19-9 (U/ml)**	> 30	148 (41.6%)	49 (41.2%)	1.000
	≤ 30	208 (58.4%)	70 (58.8%)	
**Type of operation**	Radical surgery	318 (89.3%)	98 (82.4%)	0.121
	Supplementary operation	38 (10.7%)	21 (17.6%)	
**Liver metastasis**	No	290 (81.5%)	103 (86.6%)	0.318
	Yes	66 (18.5%)	16 (13.4%)	
**Tumor infiltration depth**	Extramucosal	248 (69.7%)	83 (69.7%)	1.000
	Intramucosal	108 (30.3%)	36 (30.3%)	
**Tumor differentiation**	Poor/Moderate-to-Poor	166 (46.6%)	72 (60.5%)	**0.026**
	Good/Moderate	190 (53.4%)	47 (39.5%)	
**Neural invasion**	No	270 (75.8%)	73 (61.3%)	**0.011**
	Yes	86 (24.2%)	46 (38.7%)	
**Vascular invasion**	No	262 (73.6%)	85 (71.4%)	0.781
	Yes	94 (26.4%)	34 (28.6%)	
**T**	T1&T2	218 (61.2%)	59 (49.6%)	0.062
	T3&T4	138 (38.8%)	60 (50.4%)	
**N**	N0	222 (62.4%)	80 (67.2%)	0.463
	N1&N2	134 (37.6%)	39 (32.8%)	

**M**	M0	320 (89.9%)	111 (93.3%)	0.422
	M1	36 (10.1%)	8 (6.7%)	
**Complications**	No	262 (73.6%)	87 (73.1%)	1.000
	Yes	94 (26.4%)	32 (26.9%)	
**Postoperative infection**	No	278 (78.1%)	104 (87.4%)	0.060
	Yes	78 (21.9%)	15 (12.6%)	

Abbreviations: GBC, gallbladder cancer; PSM, Propensity Score Matching.

Table S2. Baseline characteristics of GBC patients after PSM analysis.

**Characteristics**		**Overall**	**Center 1**	**Center 2**	***P***
		**248**	**149**	**99**	
**Age (Year)**	> 60	141 (56.9%)	84 (56.4%)	57 (57.6%)	0.955
	≤ 60	107 (43.1%)	65 (43.6%)	42 (42.4%)	
**Gender**	Female	153 (61.7%)	92 (61.7%)	61 (61.6%)	1.000
	Male	95 (38.3%)	57 (38.3%)	38 (38.4%)	
**BMI**	> 28	44 (17.7%)	25 (16.8%)	19 (19.2%)	0.751
	≤ 28	204 (82.3%)	124 (83.2%)	80 (80.8%)	
**Metabolic syndrome**	No	210 (84.7%)	127 (85.2%)	83 (83.8%)	0.905
	Yes	38 (15.3%)	22 (14.8%)	16 (16.2%)	
**CA19-9 (U/mol)**	> 30	104 (41.9%)	67 (45.0%)	37 (37.4%)	0.291
	≤ 30	144 (58.1%)	82 (55.0%)	62 (62.6%)	
**Type of operation**	Radical surgery	217 (87.5%)	130 (87.2%)	87 (87.9%)	1.000
	Supplementary operation	31 (12.5%)	19 (12.8%)	12 (12.1%)	
**Liver metastasis**	No	204 (82.3%)	125 (83.9%)	79 (79.8%)	0.511
	Yes	44 (17.7%)	24 (16.1%)	20 (20.2%)	
**Tumor infiltration depth**	Extramucosal	173 (69.8%)	111 (74.5%)	62 (62.6%)	0.064
	Intramucosal	75 (30.2%)	38 (25.5%)	37 (37.4%)	
**Tumor differentiation**	Poor/Moderate-to-Poor	128 (51.6%)	76 (51.0%)	52 (52.5%)	0.917
	Good/Moderate	120 (48.4%)	73 (49.0%)	47 (47.5%)	
**Neural invasion**	No	176 (71.0%)	102 (68.5%)	74 (74.7%)	0.354
	Yes	72 (29.0%)	47 (31.5%)	25 (25.3%)	
**Vascular invasion**	No	180 (72.6%)	113 (75.8%)	67 (67.7%)	0.206

	Yes	68 (27.4%)	36 (24.2%)	32 (32.3%)	
**T**	T1&T2	136 (54.8%)	82 (55.0%)	54 (54.5%)	1.000
	T3&T4	112 (45.2%)	67 (45.0%)	45 (45.5%)	
**N**	N0	160 (64.5%)	102 (68.5%)	58 (58.6%)	0.146
	N1&N2	88 (35.5%)	47 (31.5%)	41 (41.4%)	
**M**	M0	226 (91.1%)	135 (90.6%)	91 (91.9%)	0.898
	M1	22 (8.9%)	14 (9.4%)	8 (8.1%)	
**Complications**	No	181 (73.0%)	112 (75.2%)	69 (69.7%)	0.421
	Yes	67 (27.0%)	37 (24.8%)	30 (30.3%)	
**Postoperative infection**	No	205 (82.7%)	121 (81.2%)	84 (84.8%)	0.568
	Yes	43 (17.3%)	28 (18.8%)	15 (15.2%)	

Abbreviations: GBC, gallbladder cancer; PSM, Propensity Score Matching.

Table S3. Results of ROC analysis for inflammatory biomarkers.

Factors	Cutoff	AUC (95% CI)	Specificity	Sensitivity
**NLR**	3.5	0.646 (0.504-0.840)	0.718	0.731
**PLR**	100	0.713 (0.646-0.804)	0.752	0.791
**PNI**	50	0.667 (0.446-0.816)	0.792	0.647
**LMR**	3.0	0.578 (0.478-0.746)	0.845	0.589

ROC, receiver operating characteristic; AUC, area under the curve; CI, confidence interval; NLR, neutrophil to lymphocyte ratio; PLR, platelet to lymphocyte ratio; PNI, prognostic nutritional index; LMR: lymphocyte-to-monocyte ratio.
